# Supplementary material for: Up-Cycling Grape Pomace through Sourdough Fermentation: Characterization of Phenolic Compounds, Antioxidant Activity, and Anti-Inflammatory Potential
Source: Antioxidants (Basel). 2023 Jul 29;12(8):1521. doi: 10.3390/antiox12081521 (PMC10451973; doi:10.3390/antiox12081521)
Supplement: Supplementary file 1 [file antioxidants-12-01521-s001.zip › antioxidants-2533029-supplementary.pdf]

Supplementary Material

Table S1. Bread recipes. cY-B, control bread; SD0-B, control sourdough bread, containing sourdough without grape pomace (SD0); SD5-B, bread containing sourdough added of 5% grape pomace (SD5).

% (w/w)	**cY-B**	**SD0-B**	**SD5-B**
Water	37.5	28.13	28.13
Wheat flour	62.5	46.87	46.87
SD5	0	0	25
SD0	0	25	0
Baker's yeast	2	2	2

Table S2. Nutritional characteristics of the breads: cY-B, control bread, leavened with 2% w/w baker's yeast; SD0-B, control sourdough bread, containing 25% w/w of the SD0 sourdough, and leavened with 2% w/w baker's yeast; SD5-B, a sourdough bread containing 25% w/w of the SD5 sourdough, and leavened with 2% w/w baker's yeast.

	**cY-B**	**SD0-B**	**SD5-B**
**Moisture (%)**	27.2 ± 1.3^{a}	27.4 ± 2.0^{a}	27.1 ± 1.1^{a}
**Proteins (%)**	8.68 ± 0.38^{a}	8.68 ± 0.48^{a}	8.71 ± 0.45^{a}
**Lipids (%)**	0.87 ± 0.10^{a}	0.87 ± 0.08^{a}	0.95 ± 0.09^{a}
**Carbohydrates (%)**	61.63 ± 0.36^{a}	61.63 ± 0.42^{a}	60.05 ± 0.19^{b}
**Dietary fibers (%)**	2.17 ± 0.14^{b}	2.37 ± 0.11^{b}	2.82 ± 0.09^{a}
**Ash (%)**	0.52 ± 0.2^{a}	0.52 ± 0.1^{a}	0.60 ± 0.02^{a}
**Energy Value (kJ/100g)**	1209 ± 13^{a}	1209 ± 6^{a}	1203 ± 7^{a}

The data are the means of three independent analysis ± standard deviations (n = 3).

$^{a\text{–}b}$Values in the same row with different superscript letters differ significantly ($P < 0.05$).
